# Control Efficacy of an Endophytic *Bacillus amyloliquefaciens* Strain BZ6-1 against Peanut Bacterial Wilt, *Ralstonia solanacearum*

**DOI:** 10.1155/2014/465435

**Published:** 2014-01-12

**Authors:** Xiaobing Wang, Guobin Liang

**Affiliations:** ^1^College of Environmental Science and Engineering, Yangzhou University, Yangzhou 225009, China; ^2^Jiangsu Collaborative Innovation Center for Solid Organic Waste Resource Utilization, Nanjing 210095, China; ^3^School of Chemistry and Environmental Engineering, Jiangsu University of Technology, Changzhou 213001, China

## Abstract

We aimed to isolate and identify endophytic bacteria that might have efficacy against peanut bacterial wilt (BW) caused by *Ralstonia solanacearum*. Thirty-seven endophytic strains were isolated from healthy peanut plants in *R. solanacearum*-infested fields and eight showed antagonistic effects against *R. solanacearum*. Strain BZ6-1 with the highest antimicrobial activity was identified as *Bacillus amyloliquefaciens* based on morphology, biochemistry, and 16S rRNA analysis. Culture conditions of BZ6-1 were optimized using orthogonal test method and inhibitory zone diameter in dual culture plate assay reached 34.2 mm. Furthermore, main antimicrobial substances of surfactin and fengycin A homologues produced by BZ6-1 were analyzed by high performance liquid chromatography electrospray ionization tandem mass spectrometry. Finally, pot experiments were adopted to test the control efficiency of BZ6-1 against peanut BW. Disease incidence decreased significantly from 84.5% in the control to 12.1% with addition of 15 mL (10^8^ cfu mL^−1^) culture broth for each seedling, suggesting the feasibility of strain BZ6-1 in the biological control of peanut plants BW.

## 1. Introduction

Bacterial wilt (BW) is a collective term for wilt diseases caused by at least 15 bacterial species [1]. *Ralstonia solanacearum* Smith were main strains causing wilt disease of crop plants [2] and considered as the predominant cause of peanut BW, thus negatively affecting peanut production [3].

Long-term use of chemicals to control soil-borne bacterial disease such as BW will inevitably increase peanut production cost and result in heavily environmental contamination at the same time [4]. Accordingly, approaches such as rotation, soil solarization, and deep tillage have been used to reduce chemical use, production cost, and soil contamination [5–7]. However, these strategic managements have proved less applicable in countries with limited arable land [8]. Alternatively, developing BW-resistant cultivars were regarded as the promising approach.

During the past decades, much research has been conducted on screening and breeding BW-resistant cultivars [9, 10]. It was documented that these antagonistic microorganisms showed a significant effect against BW [11, 12], and antimicrobial substances produced by bacteria were isolated and identified [13]. Most bacteria conferring resistance on cultivars belonged to rhizospheric microorganisms, which have to compete for nutrition with or directly inhibited by indigenous microorganisms in soil. As a result, these rhizospheric microorganisms can be easily affected by environmental factors and the resistant characteristics of cultivars will be lost [14]. Additionally, certain resistant cultivars were obtained by genetic modification [15, 16]. However, no genetic resources of resistance can protect vascular system from infection [17]. Therefore, other manages including biocontrol using the endophytic bacteria have been considered [18]. The advantages to use endophytes as biocontrol agents are that they are well adapted to live inside the plants and therefore they can provide reliable suppression of vascular disease [19] and do not cause environmental contamination [20]. Generally, the endophytic bacteria benefit the host plants by production of phytohormones, solubilizar phosphate, flavonoid like and antibiotic compounds, or suppressing phytopathogens by competence of invasion sites [21–23]. Recently, a lots of endophytic strains have been isolated from healthy plants [24, 25], but few have been studied from peanut plants.

Accordingly, in this paper, we aimed to isolate, screen, and identify from peanut plants the endophytic bacteria that would be effective against *R. solanacearum* and to optimize the culture conditions of the isolated strain, analyze antimicrobial substances, and test the control efficiency against peanut BW.

## 2. Material and Methods

### 2.1. Microorganisms and Cultivation

*Ralstonia solanacearum* strain used in this study was provided by Nanjing Agricultural University. It was cultured on YGPA medium containing 10 g L^−1^ of glucose, 5 g L^−1^ of peptone, 5 g L^−1^ of yeast extract, and 1 g L^−1^ of casein. The isolated entophytic bacteria were inoculated in Luria-Bertani (LB) medium containing 10 g L^−1^ of peptone, 5 g L^−1^ of yeast extract, and 5 g L^−1^ of NaCl [26].

Temperature, initial pH, and agitation speed for controlling dissolved oxygen (DO) levels were fixed at 28°C, 7.0, and 180 rpm for endophytic bacteria and 30°C, 7.2, and 200 rpm for *R. solanacearum* culture in shaking incubator, respectively.

### 2.2. Isolation and Screening of Endophytic Bacteria

The endophytic bacteria were isolated from healthy peanut plants grown in *R. solanacearum*-infested fields located at the Red Soil Ecological Experimental Station, Chinese Academy of Sciences, in Yingtan, Jiangxi Province, China. Thirty days after sowing, three healthy peanut plants were selected and taken to the laboratory for experimentation. Stems were cut off and washed twice with sterilized water, the epidermis was removed, and tissues were cut into 5 × 5 mm pieces. These were dipped in 75% alcohol for 1 min and 0.1% mercury dichloride (HgCl_2_) for 2 min, washed three times with sterilized water, mixed with 10 mL sterilized water, and ground in a chilled mortar. After 30 min precipitation, 0.1 mL of supernatant was taken, spread on LB solid media in a petri dish, and cultured in an incubator at 28°C for 72 h. Meanwhile, 0.1 mL of elution (from the third wash above) was used in the above procedure. If no colony appeared from the elution, it indicated that colonies from the supernatant belonged to endophytes. Colonies with different morphologies were picked up, microscopically examined, and purified on plates. Then, the purified cells were transferred to 50 mL fresh LB medium in a 250 mL flask and cultured in a shaker at 28°C and 180 rpm for 24 h. Afterwards, 0.1 mL cell suspensions were spread on plates and cultured in an incubator at 28°C for 72 h. In this way, further step-wise transfers were made three times. After 24 h cultivation, 40 mL medium was taken and cells were harvested by centrifugation at 10,000 rpm for 15 min and preserved at 4°C in refrigerator. Meanwhile, 0.1 mL cell suspensions were taken from the remaining 10 mL medium and inoculated on plates to screen for endophytes with antagonistic effect by using dual culture plate method [27]. That is, LB plates were prepared by mixing 0.1 mL *R. solanacearum* cells suspension (10^9^ cfu mL^−1^) with cooled and molten LB agar (42°C). The agar suspension was then dispensed into Petri dishes and was spot inoculated with test strain from 24 h culture. After cultivation in an incubator at 28°C for 72 h, those with a significant inhibitory zone were selected for further experiments.

### 2.3. Identification of Strain BZ6-1

The morphological property of BZ6-1 was examined by light microscopy and transmission electron microscopy (TEM). The biochemical and physiological characteristics were analyzed using routine methods [28]. Sequences of 16S rRNA were amplified from chromosomal DNA by PCR using universal oligonucleotide primers [29]. The primers used for amplifying and sequencing were: 8F (5′-AGAGTTTGATCCTGGCTCAG-3′) and 1541R (5′-AAGGAGGTGATCCAGCCGCA-3′). Sequences were then compared with 16S rRNA sequences in the GenBank database using BLASTN. Multiple sequence alignment was done using ClustalX 1.8 software package (http://www-igbmc.u-strasbg.fr/BioInfo/clustalx) and a phylogenetic tree was constructed by the neighbor-joining method using MEGA (Version 3.1) software. The confidence level of each branch (1,000 repeats) was tested by bootstrap analysis.

### 2.4. Assay of BZ6-1 Antimicrobial Activity

The antimicrobial activity of BZ6-1 was determined following the method of Li and Jiang [30] with minor modifications. Initially, a thin layer of agar-solidified YGPA medium (5 mL) was made in a Petri dish. Then three empty Oxford cups (stainless tube with 6 mm in inner diameter, 8 mm in outer diameter, and 10 mm in height) were placed on the surface of the medium, and 10 mL of YGPA solid medium was mixed with 0.5 mL of *R. solanacearum* cell suspension (10^9^ cfu mL^−1^) and poured to make a second thick layer. After cooling down, the Oxford cups were taken out and into each hole created by the cups, we added 0.2 mL of BZ6-1 cells suspension (10^8^ cfu mL^−1^). Dishes were incubated at 28°C for 72 h to measure inhibitory zone diameter (IZD).

### 2.5. Colonization Ability of BZ6-1 in Peanut Plants

Colonization ability of BZ6-1 in peanut plants was tested by the rifampicin-resistant method [31]. BZ6-1 was initially domesticated to grow in LB medium containing 300 *μ*g mL^−1^ of rifampicin. Peanut plants were sprayed (areas around root) with 10 mL of adapted BZ6-1 cell suspension (10^8^ cfu mL^−1^) at the seedling stage (40 days after sowing). Leaves, stems, and roots of inoculated peanut plants were collected (30 days after inoculation) to identify the endophytic bacteria (using the procedure described in Section 2.2). Supernatant was spread on solid LB medium containing 100 *μ*g mL^−1^ of rifampicin in Petri dish. The treatment without addition of BZ6-1 cell suspension was the control.

### 2.6. Design of the Orthogonal Matrix Method

BZ6-1 culture conditions were optimized using the orthogonal test based on the method [32]. The orthogonal matrix L16 (4^5^) was chosen and three factors, pH, temperature, and dissolved oxygen (DO), were arranged in the first three columns. All experiments were conducted in flasks, and culture periods were 36 h. Optimized culture conditions in flasks were also used for the 5 L fermentor with a working volume of 3 L. The pH was controlled automatically by addition of 3 mM of H_2_SO_4_ or NaOH solutions. The aeration was maintained at 1 vvm and agitation speed and temperature were adjusted automatically.

### 2.7. Purification of Antimicrobial Substances from BZ6-1

Antimicrobial substances from BZ6-1 were purified following the method [33] with minor modifications. After 24 h cultivation, 50 mL culture broth was centrifuged at 10,000 g for 10 min. After removal of cell pellets, the supernatant pH was adjusted to 2 by adding 3 mM of HCl solution, settled overnight, and centrifuged at 10,000 g for 10 min. Precipitates were collected, washed with acidified water (pH 2), neutralized by adding 3 mM of NaOH solution, freeze-dried, and dissolved in dilute NaOH solution to form a foamy liquid. After filtration through Whatman number 4 micropore membranes (20–25 *μ*m pore diameter), liquids were acidified, centrifuged, and desiccated to obtain purified antimicrobial substances.

### 2.8. Analysis of BZ6-1 Antimicrobial Substances by HPLC/ESI/CID

Purified antimicrobial substances were separated by high performance liquid chromatography (HPLC) using a reversed phase C18 analytical column (ODS: 4.6 × 250 mm, Agilent, Santa Clara, CA) with mobile phases of 0.05% trifluoroacetic acid in acetonitrile (solvent A) and 0.05% trifluoroacetic in Milli-Q water (solvent B) at a flow rate of 200 *μ*L min^−1^ using a gradient elution with UV detection at 210 nm. The elution conditions were 50–100% (A), 0–50 min, 50–0% (B), 0–50 min; 100% (A), 50–70 min, 0% (B) 50–70 min. Electrospray ionization/collision induced dissociation (ESI/CID) mass spectrometry was performed using a Surveyor-LCQ DECA XP Plus (Thermo Finnigan, San Jose, CA, USA). The electrospray source was operated at a capillary voltage of 15 V, a spray voltage of 5 kV, and a capillary temperature of 275°C. Helium was used as the collision gas for the CID experiment and the collision energy was set at 35% [34].

### 2.9. BZ6-1 Biocontrol Effect in Pot Experiments

Four treatments were designed to test BZ6-1 resistance to *R. solanacearum* in pot experiments. Each treatment consisted of ten pots and each pot was 40 cm in diameter and filled with 15 kg of equal quantities of paddy and dry soil. In each pot, three peanut seedlings were planted. Each treatment had four replications and was repeated twice. Two weeks after sowing, healthy seedlings with a similar size were selected and transplanted to pots. Three days after transplantation, each seedling was treated with 10 mL *R. solanacearum* cell suspensions (10^9^ cfu mL^−1^). After two weeks growing in pots, per seedling of treatment was inoculated with 5, 10, 15, or 20 mL of BZ6-1 cells suspension (10^8^ cfu mL^−1^) with about 80% cells in total containing intracellular spores for each seedling. Healthy peanut plants were numbered to calculate disease incidence during the fruiting period. Disease incidence is expressed as the ratio of BW-seedlings to total plants numbers multiplied by 100%. A pot without addition of BZ6-1 cell suspension was the control. All pots experiments were conducted in greenhouse and water content in pot soil was kept about 70% by watering periodically.

### 2.10. Statistical Analysis

All experiments were conducted in triplicate and repeated twice and the average of the results was used for analysis. Quantitative data is expressed as means ± standard deviations and was analyzed by one-way ANOVA using the Statistical Package for Social Sciences (SPSS) 13.0 for Windows. Multiple comparisons between groups were performed using the Student-Newman-Keuls (SNK) analysis test. Statistical significance was set at a confidence level of *P* < 0.05.

## 3. Results

### 3.1. Isolation and Screening of Endophytic Bacteria Resistant to R. solanacearum

Initially, epiphytic parts of the peanut plants were disinfected by alcohol and mercury dichloride (HgCl_2_) to get rid of bacteria on epidermis. Then, to check whether the strains isolated from peanut plants were endophytic bacteria or not, elutions from the third wash of leaves or stems were inoculated onto plates. Results showed that no colony occurred, preliminarily indicating that isolated strains belonged to endophytic bacteria. Based on morphological differences, a total of 37 strains were isolated from healthy peanut plants grown in *R. solanacearum*-infested fields. Additionally, inhibitory zone diameter (IZD) was used to study the antimicrobial effects of isolated strains on *R. solanacearum*. It was observed that in eight strains the IZD exceeded 10 mm (Table 1) and that of BZ6-1 reached 16.4 mm, suggesting the highest antimicrobial activity. To further document that BZ6-1 is an endophytic bacterium, its colonization ability of peanut was tested. The endophytic bacteria isolated from leaves, stems, and roots of peanut plants were the same as BZ6-1 in morphology, physiology, and biochemistry and in antagonism to pathogenic bacteria, suggesting that BZ6-1 is a typical endophytic bacterium. In addition, colony numbers of 3.50 × 10^5^ cfu/g fresh weight in roots, 6.60 × 10^4^ cfu/g in stem, and 4.20 × 10^4^ cfu/g in leaf indicated that BZ6-1 had a preferential niche of colonization in roots. Accordingly, BZ6-1 was selected as an experimental strain for our following work.

### 3.2. Morphological and Biochemical Characteristics and 16S rRNA Analysis of BZ6-1

**Figure 1:**
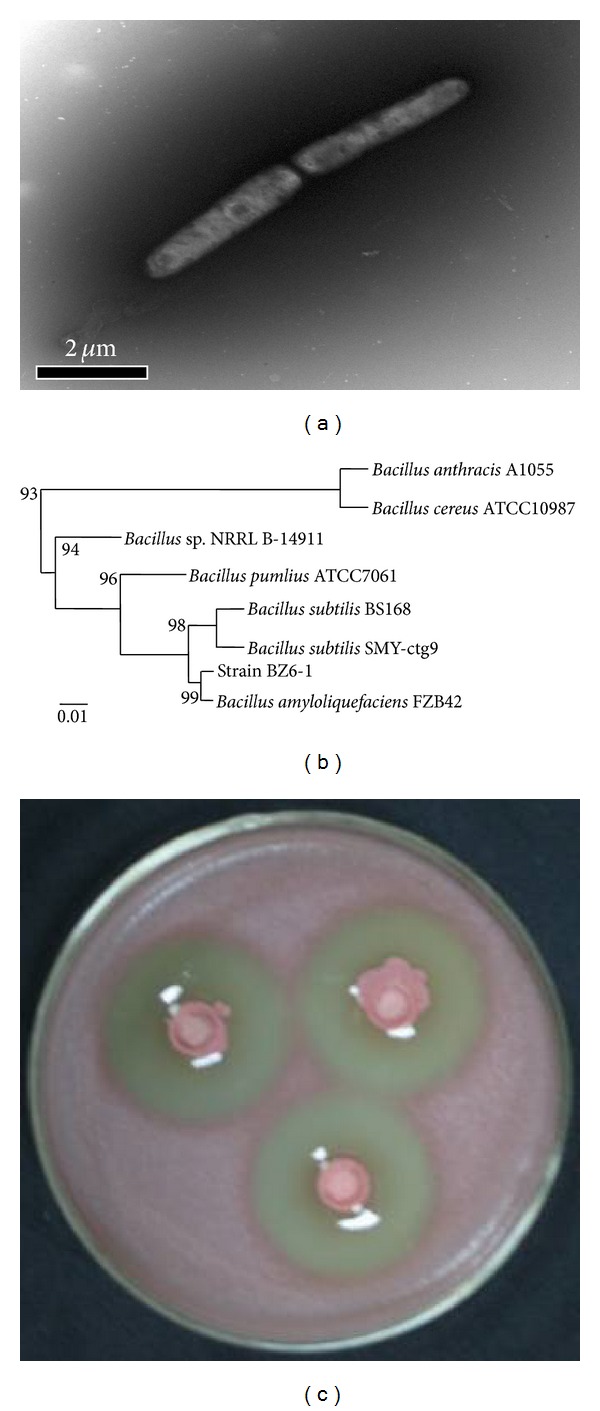
(a) Transmission electron microscopy (TEM) of BZ6-1 cell morphology with negative staining (×26,500). (b) Phylogenic tree including BZ6-1 based on 16S rRNA full-length sequences. (c) Inhibition zone of BZ6-1 against *R. solanacearum*.

To further identify BZ6-1, we observed morphological and biochemical characteristics and conducted 16S rRNA analysis. Colonies of strain BZ6-1 on solid LB plates were cream in color, opaque, and convex with a wrinkled surface and entire margins. Cells were Gram positive and rod shaped. A transmission electron micrograph (TEM) of BZ6-1 morphology was shown in Figure 1(a). Physiological and biochemical tests indicated BZ6-1 to be aerobic, catalase positive, nitrate reduction positive, indole positive, capable of starch and gelatin hydrolysis, methyl red negative, and Voges-Proskauer negative (data not shown). The sequenced 16S rRNA of BZ6-1 was 1,458 bp in length (data not shown). As indicated in Figure 1(b), BZ6-1 is a *Bacillus* species, most closely adjacent to *Bacillus amyloliquefaciens* (Genbank accession number NC009725) with a similarity of up to 99%. Consequently, from morphological and biochemical characteristics and 16S rRNA sequence, strain BZ6-1 was identified as *Bacillus amyloliquefaciens* BZ6-1; its Genbank accession number is JF693628.

### 3.3. Enhancement of BZ6-1 Antimicrobial Activity by Optimizing Culture Conditions

Environmental factors including pH, temperature, and dissolved oxygen (DO) can significantly affect bacterial growth and antimicrobial substances formation. The combination effects of optimum levels of each factor may not be the optimal conditions. The orthogonal method is an efficient experimental design and can avoid large amount of experiments brought by full-factors experimental projects but give a satisfactory result. Accordingly, the orthogonal matrix L16 (4^5^) was chosen to optimize culture conditions. Experimental factors and their levels for orthogonal projects were listed in Table 2(a), and conditions for each project and experimental results were indicated in Table 2(b). *K* value is the average IZD of every factor under each level. According to the largest donating rule, the largest value of *K* is the optimized value. *R* value is the range of *K* value. Cell concentrations were measured spectrophotometrically at a wavelength of 600 nm. Optical density (OD_600_) was positively proportional to IZD, indicating that the status of cell growth can indirectly reflect antimicrobial activity. In addition, according to statistical calculation, a pH of 6.5, a temperature of 25°C, and DO of 200 rpm are likely to achieve the highest cell concentration. To confirm this analyzed optimal point, experiments were carried out and results showed that OD reached 1.62 after 36 h cultivation, and the corresponding IZD was 34.2 mm (Figure 1(c)). Furthermore, according to *R* (Max. Dif.) in Table 2(b), the influence order of three factors on cell growth was pH > DO > temperature. This was further demonstrated by variance analysis (Table 2(c)). It was suggested that the effect of pH on cell growth was the most significant.

**Figure 2:**
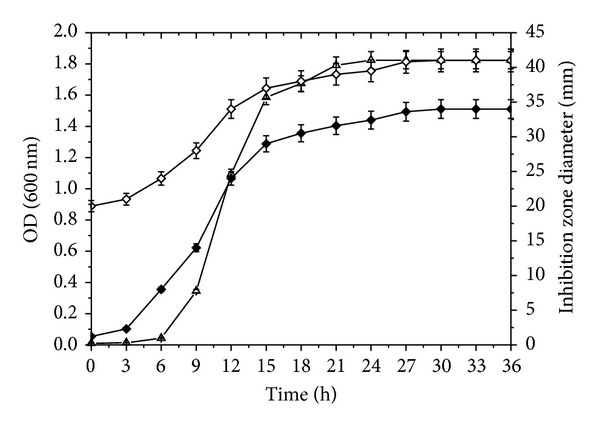
Effects of culture time on antimicrobial activity and cells growth of BZ6-1. Note: ◆: supernatant IZD; *⋄*: cell suspension IZD; △: OD.

As pH values in flasks cannot be precisely controlled, the optimal culture conditions achieved in flasks were further applied to a 5 L fermentor. Moreover, to check whether antimicrobial substances were being produced extracellularly, the supernatant and cell suspensions at different culture times were tested for antimicrobial activity. As indicated in Figure 2, with increased culture time, cell concentration increased correspondingly. After 24 h cultivation, cells stopped propagation and the OD reached its highest value (1.82), 12% higher than that in the flask experiment. It was also clear that the time profiles of IZD had similar trends to cell growth, which indicates that the quantities of antimicrobial substances being produced were positively proportional to cell concentrations. Moreover, it was observed that the IZD of cells suspension was slightly larger than that of the supernatant. As is known, in contrast to supernatants, cell suspensions can generate antimicrobial substances continuously during the process of plate cultivation. As a result, relatively higher amounts were produced, thus leading to a larger IZD in the end. However, as the differences in IZD between the two treatments were slight, we can reasonably conclude that antimicrobial substances are mainly generated inside cells and then secreted into the supernatant.

We took BZ6-1 cell suspensions from fermentor at different culture times to measure antimicrobial activity. Results showed that the highest cell suspension IZD reached 39.3 mm at 27 h, an increase of 14.6% compared with the optimal one in the flasks. In brief, the better results in cell growth and IZD obtained in fermentor can be attributed to a stable pH but are difficult to be realized in flask experiment.

### 3.4. Structural Characterization of BZ6-1 Antimicrobial Substances

**Figure 3:**
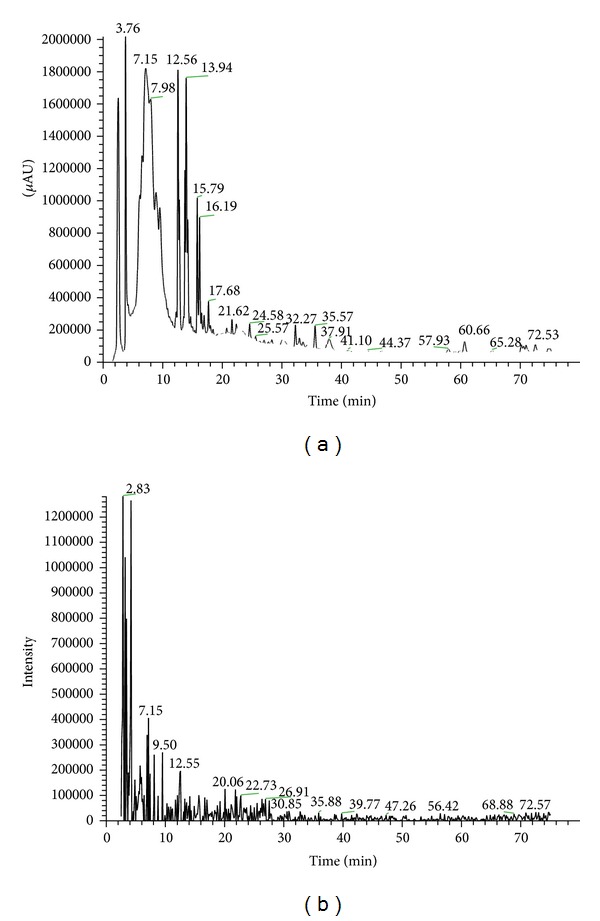
((a) and (b)) HPLC chromatogram and positive ion spectra of antibacterial substances produced by BZ6-1. Note: (a) HPLC chromatogram; (b) positive ion spectra.

**Figure 4:**
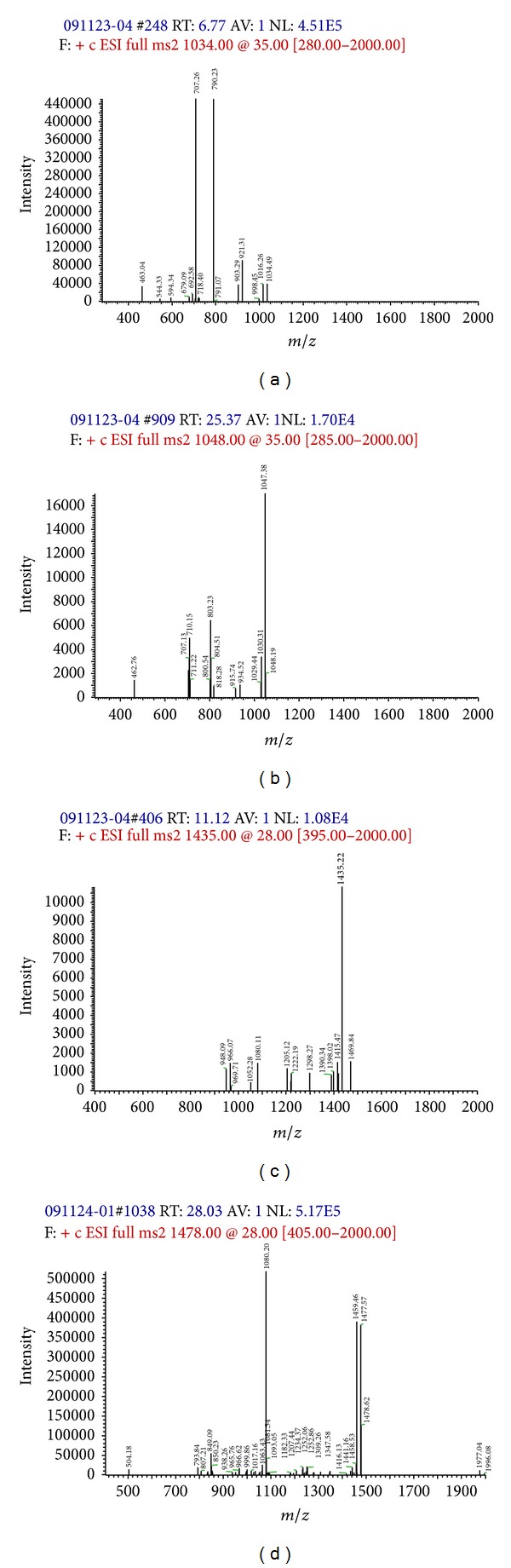
CID spectra of surfactin and fengycin A homologues produced by BZ6-1. Note: (a)–(d) are *m*/*z* 1034.5, 1048.1, 1435.2, and 1477.6, respectively.

Our results indicated that BZ6-1 can generate antimicrobial substances and show strong inhibition of *R. solanacearum*. Accordingly, these substances were initially separated by HPLC (Figure 3(a)) and the molecular mass of purified compounds was measured using ESI-MS spectrometry (Figure 3(b)). Based on molecular weights of surfactin and fengycin A homologues, four main peaks at mass to charge ratios (*m*/*z*) of 1034.5, 1048.1, 1435.2, and 1477.6 were obtained, and each of these ions was selected as a precursor ion for further CID analysis. The product ions of *m*/*z* 707 were found in the CID spectra of precursor ions of *m*/*z* 1034.5 and 1048.1 (Figures 4(a) and 4(b)). Meanwhile, product ions of *m*/*z* 966 and 1080 were found in the CID spectra of precursor ions of *m*/*z* 1435.2 and 1477.6 (Figures 4(c) and 4(d)).

### 3.5. Control Efficacy of BZ6-1 on Peanut BW in Pot Experiments

As known from the above results, BZ6-1 can generate antimicrobial substances extracellularly, thus inhibiting *R. solanacearum* growth. To further test effects of BZ6-1 on peanut BW, pot experiments were conducted. As indicated in Table 3, with an increase in the quantity of BZ6-1 added, disease incidence correspondingly decreased. In the control, for example, disease incidence was 84.5% and the corresponding value was 23.6% with addition of a 10 mL BZ6-1 cells suspension (10^8^ cfu mL^−1^) for each seedling. Moreover, the disease incidence with a 15 mL suspension addition was slightly higher than that with a 20 mL addition. It was suggested that a 15 mL suspension is suitable for control of peanut BW.

## 4. Discussion

BW, a soil-borne bacterial disease caused by *R. solanacearum*, is an important constraint to peanut production and difficult to control. The association of endophytic microorganisms and plants does not cause any visible damage but can benefit plants with different mechanisms [35]. Recent study has documented that some endophytic bacteria can generate certain antimicrobial substances that show resistance to bacterial disease [36]. However, to date, there were few reports of the isolation of endophytic bacteria from peanut in the biological control of *R. solanacearum*.

Consequently, we isolated endophytic bacteria from healthy peanut plants in this study. Thirty-seven endophytic bacteria were isolated and eight strains showed antagonistic effects on *R. solanacearum*. BZ6-1 had the highest antimicrobial activity and was therefore selected for the systematic investigation of its characteristics. BZ6-1 was found to have phenotypes and biochemical characteristics similar to *Bacillus*. However, 16S rRNA gene sequences showed limited variation in closely related species [37], and phylogenetic analysis was able to accurately classify *B. subtilis* and related taxa [38]. Accordingly, phylogenetic analysis was used to help identify BZ6-1. Based on morphology, physiology, biochemistry, and phylogenetic position from 16S rRNA analysis, BZ6-1 was identified as *B. amyloliquefaciens*. Besides BZ6-1, we will continue the research about the phylogenetic diversity of the thirty-seven endophytic bacteria and biocontrol ability of eight strains in the future study.

Statistical experimental design, as an efficient way to improve experimental works, has been widely used in chemistry, food, and environmental engineering [39]. Orthogonal test was considered as a powerful technique for testing multiple process variables and identifying interactions between variables, and a combination of multiple factors generating an optimal result can be identified by this method [40].

Medium pH, temperature, and DO concentrations are thought to be important for BZ6-1 growth and antimicrobial substances production. So, these factors were optimized using orthogonal test method. Results showed that medium pH had the most significant effect. Optimized conditions markedly promoted cell growth, leading to an increase in antimicrobial activity. A lot of reports have shown that potential biocontrol products include bacteria, culture filtrates, and even their ingredients [41, 42]. Our results suggested that antimicrobial substances were mainly released in the culture medium but rather than being associated with intact cells.

Recent publications have demonstrated that most antimicrobial substances generated by *B. amyloliquefaciens* are lipopeptides, mainly surfactin, fengycin, and iturin [43–45]. Surfactin consists of a *β*-hydroxy fatty acid and a small peptide with seven amino acid residues as follows:
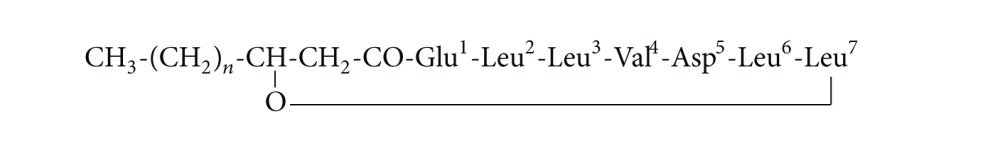
(1)


Because of different amino acids in the fourth and seventh peptide chains or different lengths of fatty acid chains, there are many surfactin homologues. Based on the seventh amino acid of the peptide chain being Leu or Val, surfactin homologues have two main types [46, 47]. The ions of *m*/*z* 707, which could be explained as [Leu-Leu-Val-Asp-Leu-Leu + Na^+^ + H_2_O], were considered surfactin “fingerprints” [34]. In addition, fengycin, with circular and linear structures and mainly composed of *β*-hydroxy fatty acids bound to ten amino acids (AA), has a series of homologous compounds and is considered typical antimicrobial substances. Circular fengycin A, for example, is symbolized by Ala, the sixth in the AA sequence with an oxygen atom forming a bridge between Tyr and Ile, as shown in following formula [48, 49]:
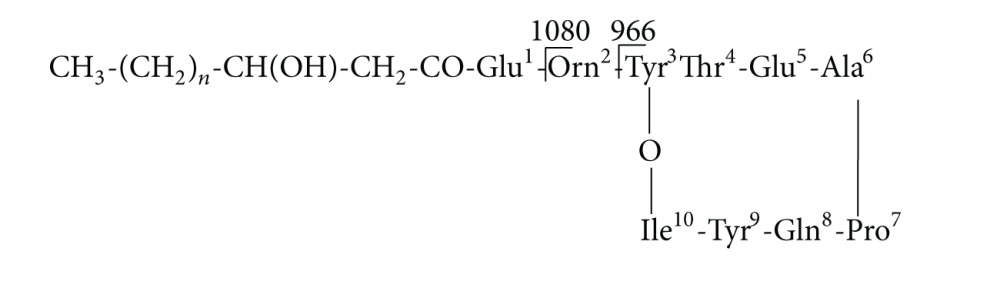
(2)


The ions of *m*/*z* 966 and 1080 can be considered “fingerprints” of fengycin A [14, 50].

Therefore, based on our results, it can be safely concluded that peaks at *m*/*z* 1034.5 and 1048.1 (Figures 4(a) and 4(b)) were protonated molecular ion peaks [M + Na + H_2_O]^+^ of surfactin homologues with C14 and C15. Meanwhile, *m*/*z* 1435.2 and 1477.6 (Figures 4(c) and 4(d)) can be explained as neutral losses of fatty acid-Glu and fatty acid-Glu-Orn, respectively, from the N-terminus segment of fengycin A. Accordingly, we preliminarily concluded that *m*/*z* 1477 and *m*/*z* 1435 belong to circular fengycin A, composed of C_17_ and C_14_ carbon units fatty acids and ten AAs. In addition, *m*/*z* 1080 and 966 can be attributed to dissociation at Glu and Orn, respectively, in circular fengycin A, and can be expressed as follows (see (3) and (4)):
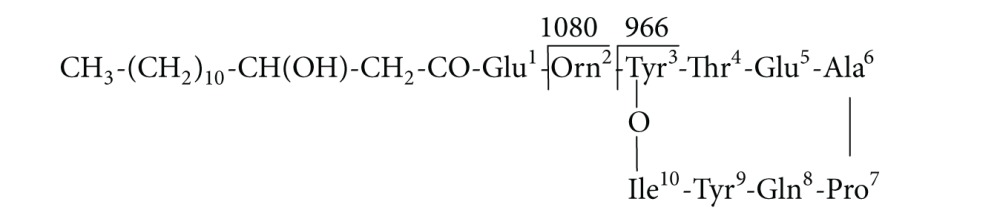
(3)

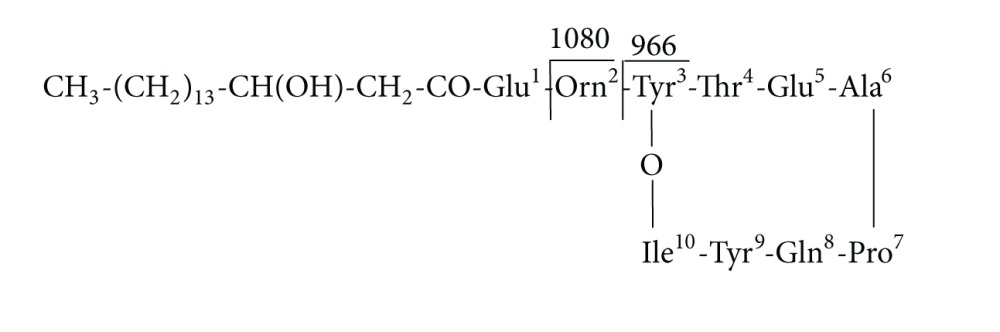
(4)


Thus, we preliminarily analyzed and identified surfactin and fengycin A homologues. Other antimicrobial substances need further identification in future work.

Finally, pot experiments were adopted to test control efficiency of BZ6-1 on peanut BW. With an increase in the quantity of cell suspension added, disease incidence correspondingly decreased (Table 3). As BW is caused by *R. solanacearum*, disease control efficiency was mainly related to quantities of antimicrobial substances being produced by BZ6-1. Decreased disease incidence that resulted from increased amounts of cell suspension was observed. It was perhaps indicated that surfactin and fengycin A homologues play key roles in controlling peanut BW evoked by *R. solanacearum*. However, other bioactive substances that may be produced by BZ6-1 need to be further investigated and identified in the future.

## Figures and Tables

**Table 1:** Antimicrobial activity of eight endophytic bacteria strains against *R. solanacearum. *

Strain	IZD (mm)
BZ-1	10.5 ± 0.2^c^
BZ-2	12.6 ± 0.3^b^
BZ-5	10.8 ± 0.4^c^
BZ6-1	16.4 ± 0.5^a^
BZ-7	11.3 ± 0.4^c^
XZ-1	10.9 ± 0.3^c^
XZ-5	12.4 ± 0.3^bc^
XZ-7	13.2 ± 0.5^b^

Note: IZD means inhibitory zone diameter. Values are means ± standard deviations. The same letters mean no significant difference at *P* < 0.05.

**Table:** (a)

Levels	pH	DO (rpm)	Temperature (°C)
1	5.5	180	20
2	6.5	200	25
3	7.5	220	30
4	8.5	240	35

Note: levels 1–4 represent concentration levels of each factor.

**Table:** (b)

Trial number	pH	DO (rpm)	Temperature (°C)	IZD (mm)	OD_600_
1	1	1	1	24.2 ± 0.4^e^	0.67 ± 0.06^d^
2	1	2	2	27.6 ± 0.3^d^	0.98 ± 0.08^c^
3	1	3	3	26.4 ± 0.6^de^	0.86 ± 0.04^cd^
4	1	4	4	24.8 ± 0.2^e^	0.65 ± 0.05^d^
5	2	1	2	30.5 ± 0.5^b^	1.23 ± 0.06^bc^
6	2	2	1	32.5 ± 0.3^a^	1.47 ± 0.03^a^
7	2	3	4	31.3 ± 0.5^b^	1.28 ± 0.04^b^
8	2	4	3	30.8 ± 0.2^b^	1.22 ± 0.05^bc^
9	3	1	3	26.6 ± 0.6^d^	0.85 ± 0.06^cd^
10	3	2	4	26.8 ± 0.5^d^	0.88 ± 0.08^cd^
11	3	3	1	25.5 ± 0.3^e^	0.76 ± 0.04^d^
12	3	4	2	28.7 ± 0.5^cd^	1.06 ± 0.05^c^
13	4	1	4	26.8 ± 0.6^d^	0.89 ± 0.03^cd^
14	4	2	3	23.5 ± 0.4^f^	0.56 ± 0.05^e^
15	4	3	2	25.8 ± 0.3^e^	0.78 ± 0.04^d^
16	4	4	1	22.4 ± 0.5^f^	0.45 ± 0.07^e^
*K*1	25.750	27.025	26.150		
*K*2	31.250	27.600	28.150		
*K*3	26.900	27.250	26.825		
*K*4	24.625	26.675	27.425		
*R*	6.650	0.925	2.000		

Note: values are means ± standard deviations. The same letters mean no significant difference at *P* < 0.05.

**Table:** (c)

Variance sources	Sum of deviation squares	Df	*F* value	Significance
pH	103.69	2	4.052	*P < 0.05
DO	2.19	2	0.086	
Temperature	0.68	2	0.261	
Error	127.94	8	0.086	

**Table 3:** Effects of different amounts of BZ6-1 cells suspension on peanut bacterial wilt.

Treatments	Control	5 mL	10 mL	15 mL	20 mL
Disease incidence (%)	84.5 ± 3.2^a^	44.6 ± 2.1^b^	23.6 ± 1.8^c^	13.3 ± 1.5^d^	12.1 ± 1.2^d^

Note: values are means ± standard deviations. The same letters mean no significant difference at *P* < 0.05.
